# Accuracy of photorespiration and mitochondrial respiration in the light fitted by CO_2_ response model for photosynthesis

**DOI:** 10.3389/fpls.2025.1455533

**Published:** 2025-08-26

**Authors:** Zhengwen Niu, Zi-Wu-Yin Ye, Qi Huang, Chunju Peng, Huajing Kang

**Affiliations:** ^1^ Wenzhou Key Laboratory of Agricultural & Forestry Carbon Sequestration and Tea Resource Development, Wenzhou Academy of Agricultural Sciences, Wenzhou, Zhejiang, China; ^2^ College of International Studies, Guangdong Baiyun University, Guangzhou, Guangdong, China

**Keywords:** CO2 concentration, CO2 recovery, mitochondrial respiration rate, photorespiration, global change

## Abstract

**Introduction:**

Atmospheric CO_2_ elevation significantly impacts plant carbon metabolism, yet accurate quantification of respiratory parameters—photorespiration rate (R_p_) and mitochondrial respiration rate in the light (R_d_)—under varying CO_2_ remains challenging. Current CO_2_-response models exhibit limitations in estimating these parameters, hindering predictions of crop responses under future climate scenarios.

**Methods:**

Low-oxygen treatments and gas exchange measurements, calculating CO_2_ recovery/inhibition ratio in of wheat (*Triticum aestivum L.*) and bean (*Glycine max L.*) were employed to elucidate the biological significance and interrelationships of R_p_ and R_d_. Model-derived estimates of R_p_ and R_d_ were compared with measured values to assess the accuracy of three CO_2_-response models (biochemical, rectangular hyperbola, modified rectangular hyperbola). Furthermore, the effects of ambient CO_2_ concentration (0~1200 μmol·mol^-1^) on the measured R_p_ and R_d_ were quantified through polynomial regression.

**Results:**

The A/C_a_ model achieved superior fitting performance over the A/Ci model. However, significant disparities persisted between A/Ca-derived R_p_/R_d_ estimates and measurements (*p* < 0.05). CO_2_ concentration exhibited dose-dependent regulation of respiratory fluxes: R_p-measured_ ranged from 4.923 ± 0.171 to 12.307 ± 1.033 μmol (CO_2_) m^-2^ s^-1^ (wheat) and 4.686 ± 0.274 to 11.673 ± 2.054 μmol (CO_2_) m^-2^ s^⁻ ¹^ (bean), while R_d-measured_ varied from 0.618 ± 0.131 to 3.021 ± 0.063 μmol (CO_2_) m^-2^ s^-1^ (wheat) and 0.492 ± 0.069 to 2.323 ± 0.312 μmol (CO_2_) m^-2^ s^-1^ (bean). Polynomial regression revealed strong non-linear correlations between CO_2_ concentrations and respiratory parameters (R^²^ > 0.891, *p* < 0.05; except bean R_p-_C_a_: R^²^ = 0.797). Species-specific CO_2_ thresholds governed peak R_p_ (600 μmol·mol^-1^ for wheat vs. 1,000 μmol·mol^-1^ for bean) and R_d_ (400 μmol·mol^-1^ for wheat vs. 200 μmol·mol^-1^ for bean).

**Discussion:**

These findings expose critical limitations in current respiratory parameter quantification methods and challenge linear assumptions of CO_2_-respiration relationships. They establish a critical framework for refining photosynthetic models by incorporating CO_2_-responsive respiratory mechanisms. The identified non-linear regulatory patterns and model limitations provide actionable insights for advancing carbon metabolism theory and optimizing crop carbon assimilation strategies under rising atmospheric CO_2_, with implications for climate-resilient agricultural practices.

## 1 Introduction

Atmospheric CO_2_ concentration has increased by 48% since the pre-industrial era, reaching 415 ppm in 2021 (IPCC, 2021). This elevation drives dual climate-ecosystem impacts: as a primary greenhouse gas contributing to global warming through radiative forcing, and as a photosynthetic substrate enhancing plant productivity via the “CO_2_ fertilization” effect (Easterling et al., 2000; Vaughan et al., 2003). However, the physiological mechanisms underlying plant adaptation to elevated CO_2_—particularly regarding respiratory metabolism—remain insufficiently quantified (Xanthopoulos et al., 2017; Jalali et al., 2020).

Photosynthesis, transpiration, and respiration are three vital processes in plant life, essential for growth and metabolism (Nilsen, 1995). Transpiration facilitates water transport across the soil-plant-atmosphere continuum (SPAC), supporting plant growth and influencing ecosystem water-heat balances (Liu and Yu, 1997). Photosynthesis allows plants to convert light energy, CO_2_, and water into organic matter and oxygen, directly impacting the productivity of terrestrial ecosystems and the global carbon cycle (Schimel et al., 2001). Photorespiration, however, occurs when the photosynthetic enzyme Rubisco reacts with oxygen instead of CO_2_—a common scenario under high temperature, drought, or intense light that limits CO_2_ availability. This process, universal in oxygen-producing plants and algae (Carvalho et al., 2011), shares chloroplasts as the primary site with photosynthesis and relies on light-driven reactions. Crucially, photorespiration recycles harmful byproducts generated during photosynthesis, balancing energy use and protecting plants from stress, thereby acting as a “safety valve” for photosynthetic efficiency. Mitochondrial respiration involves oxidative phosphorylation in the cells’ mitochondria to produce energy for vital activities (Vercellino and Sazanov, 2022; Huang et al., 2023). These biological processes adapt to elevated CO_2_ concentrations and climate warming (Luo et al., 2001), affecting carbon cycling processes in terrestrial ecosystems (Lin, 1998; Zhang et al., 2000; Wang et al., 2008). Thus, understanding how crop photorespiration and mitochondrial respiration respond to changes in atmospheric CO_2_ is crucial for predicting future crop productivity and growth patterns under elevated CO_2_ conditions.

Wheat and bean are globally significant crops, crucial to human food supply and agricultural production (Gan et al., 2015; Borrell et al., 2017). As typical C_3_ plants, CO_2_ is the main limiting factor affecting their photosynthesis (Atkin et al., 2005). Traditional theories suggest that the carbon source for photosynthesis in terrestrial plants is primarily atmospheric CO_2_, often neglecting CO_2_ released by photorespiration and dark respiration of the leaves (Stirbet et al., 2020). And related respiration parameters (photorespiration rate (R_P_), mitochondrial respiration rate in the light (R_d_), and respiration in the light (R_L_)) are inconsistently applied (von Caemmerer, 2000). The chloroplast interior constitutes the primary site for photorespiratory CO_2_ release, where a substantial proportion of respired CO_2_ undergoes re-assimilation through the Calvin cycle. This CO_2_ recycling mechanism plays a crucial physiological role by enhancing subcellular CO_2_ concentration independent of diffusion limitations imposed by boundary layer resistance stomatal conductance, and mesophyll resistance (Loreto et al., 1999, 2001; Pinelli and Loreto, 2003). Quantitatively studying photorespiratory CO_2_ recycling in C_3_ plants is challenging due to the simultaneous occurrence of photorespiration, dark respiration, and photosynthetic carbon assimilation within mesophyll cells (Haupt-Herting et al., 2001). The carbon isotope method can distinguish between CO_2_ fixed by photosynthesis and released by photorespiration, offering a potential approach to studying CO_2_ recycling and reuse by plants (Ostle et al., 2000). However, this method is costly, complex, and not precise in exploring photorespiratory CO_2_ recycling, because this method ignores two important factors: firstly, Rubisco’s affinity for ^14^CO_2_ and ^13^CO_2_ is much lower than that of ^12^CO_2_; secondly, the measurement process inevitably involves the inhibition of photorespiration by extremely high concentrations of ^12^CO_2_ (30000 μmol mol^-1^) (Pärnik and Keerberg, 2007; Busch and Sage, 2017). Kang et al. (2014) used gas exchange methods to confirm that CO_2_ released by light and dark respiration can be reutilized by photosynthesis, though this concept is often overlooked. Therefore, accurate estimation of photorespiratory CO_2_ reutilization is essential for improving the accuracy of photosynthetic parameters and carbon metabolism processes.

The CO_2_ response curve for photosynthesis is a valuable tool for studying plant physiology and ecology, providing insights into how photosynthetic properties respond to environmental factors. This understanding can optimize CO_2_ concentration management in agricultural production, contributing to enhance photosynthetic efficiency and promote growth and productivity. Two types of CO_2_ response models exist: biochemical and empirical. The most widely used biochemical model is the Farquhar model and its modifications (von Caemmerer and Farquhar, 1981; Bernacchi et al., 2001; Long and Bernacchi, 2003; Ethier and Livingston, 2004). Empirical models include the rectangular hyperbola model (Ye, 2010), modified version (Ye and Yu, 2009), and the Michaelis-Menten model (Harley et al., 1992). However, current biochemical models do not consider the effect of mitochondrial respiration rate in the light (R_d_), and empirical models overlook the effect of CO_2_ concentration on photorespiration rate. Quantitative studies on the effect of atmospheric CO_2_ concentration (C_a_) on R_p_ and R_d_ are limited, making the accuracy of R_p_ and R_d_ values from current CO_2_ response models uncertain.

Addressing these research gaps, this study used low oxygen and gas exchange methods, with calculating CO_2_ recovery and inhibition ratio, aiming to: (1) elucidate the biological significance and interrelationships of photosynthetic parameters related to light and dark respiration, and accurately measure or calculate them; (2) compare R_p_ and R_d_ values between fitted and measured data to evaluate the CO_2_ photosynthetic response model’s accuracy, identifying a more precise estimation model (A/C_a_ or A/C_i_); and (3) provide quantitative descriptions of C_a_ or C_i_ *(*depends on the accuracy of the model) effects on R_p-measured_ and R_d-measured_, offering theoretical research insights for the practical application of photosynthetic carbon metabolism processes and the promotion of carbon metabolism.

## 2 Materials and methods

### 2.1 CO_2_ response models

The biochemical model can be expressed as


(1) $$P_{n}=min\left\{w_{c}\;,\;w_{j}\;,\;w_{p}\right\}\left(1-\frac{\tau^{*}}{C_{i}}\right)-R_{d}$$

where *P_n_* is the net photosynthetic rate; and *w*_c_, *w_j_* and *w_p_* are the potential rates of CO_2_ assimilation that can be supported by the enzymes of ribulose 1,5-bisphosphate (RuBP) carboxylase/oxygenase (Rubisco), RuBP- regeneration and triose-phosphate utilization, respectively. The photosynthetic compensation point ( $\tau^{*}$ ) is the CO_2_ concentration at which the photorespiratory efflux of CO_2_ equals the rate of photosynthetic CO_2_ uptake. *C_i_* is the intercellular CO_2_ concentration and *R_d_* is the mitochondrial respiration rate in the light.

Empirical models include the rectangular hyperbola model, modified version, and the Michaelis-Menten model, as following:

The rectangular hyperbola model can be represented as


(2) $$P_{n}=\frac{aP_{nmax}C_{i}}{aC_{i}+P_{nmax}}-R_{p}$$

where *P_n_*_max_ is the maximum photosynthetic rate; *R_p_* is the photorespiration rate (in fact this parameter represents the respiration rate in the light (*R_L_*), which includes *R_p_* and *R_d_*. *A* detailed description is given in the text.); and α is the initial slope of the CO_2_ response curve; *C_i_* is the same as above.

The Michaelis−Menten model can be displayed as


(3) $$P_{n}=\frac{P_{nmax}C_{i}}{C_{i}+K}-R_{p}$$

where *P_n_*_max_, *R_p_*, *P_n_* and *C_i_* are the same as above, and *K* is the Michaelis-Menten constant.

The modified rectangular hyperbola model can be written as


(4) $$P_{n}=a\frac{1-bC_{i}}{1+cC_{i}}C_{i}-R_{p}$$

where α, *R_p_*, *P_n_* and *C_i_* are the same as above, and *b* and *c* are coefficients (mol μmol^–1^) (Ye, 2010) (Equations 1–4).

According to this equation, the Michaelis-Menten and the rectangular hyperbola model are essentially the same. Therefore, the fitted results for the Michaelis-Menten model were not shown in this paper.

### 2.2 Theoretical considerations

#### 2.2.1 Calculation of photorespiration rates

R_p_ were determined through differential gas exchange measurements under contrasting O_2_ concentrations. Measurements were conducted using a LI-6400XT portable photosynthesis system (LI-COR Biosciences, Lincoln, NE, USA) with the following standardized conditions:

1. Ambient O_2_ treatment (21% O_2_): At ambient CO_2_ (0 μmol·mol^−1^) and saturating Photosynthetically Active Radiation (PAR) (2000 μmol·m^−2^·s^−1^), the net photosynthetic rate (P_n21%_) represents combined respiratory fluxes (R_L_ = R_p_ + R_d_), as photorespiration proceeds normally while CO_2_ in photosynthesis comes from respiration.
2. Low O_2_ treatment (2% O_2_): Under identical CO_2_ and light conditions, complete inhibition of photorespiration in wheat and bean leaves was achieved based on our previous validation (Kang et al., 2014). The measured P_n2%_ thus corresponds specifically to R_d_.

R_p_ was calculated using the respiratory partitioning equation (Erdei et al., 2001; Laisk et al., 2002; Parys et al., 2004):


(5) $$\begin{array}{c} R_{p}=P_{n2\%}-P_{n21\%} \end{array}$$

where *P_n2%_* and *P_n_*_21%_ are the photosynthetic rates at 2% O_2_ and 21% O_2_, respectively.

#### 2.2.2 Calculation of CO_2_ recovery and inhibition ratios of photorespiration

Our prior mechanistic studies revealed concentration-dependent regulation of photorespiratory CO_2_ recovery. The photorespiratory CO_2_ recovery ratio (R_pe-i_), defined as the proportion of respired CO_2_ re-assimilated by chloroplasts, decreased progressively with increasing ambient CO_2_ concentration. Beyond threshold CO_2_ concentrations, competitive inhibition between photorespiration and carboxylation pathways significantly suppressed photorespiratory flux (Kang et al., 2013). *R_pe-i_* and photorespiratory inhibition index (*I_i_*) can be quantified through Equation 6.


(6) $$\begin{array}{c} R_{pe-i}\;or\;I_{i}=\frac{R_{pmax}-R_{p-i}}{R_{pmax}} \end{array}$$

where $\;R_{pe-i}\;$ and $I_{i}$ are the CO_2_ recovery ratio and inhibition ratio of photorespiration, *R_p_*_max_ is the maximum photorespiration rate and *R_p_*_−_ *_i_* is the photorespiration rate at *i* CO_2_ concentrations, *i* represents different CO_2_ concentrations.

#### 2.2.3 Calculation of the mitochondrial respiration rates in the light

Both mitochondrial respiration-derived CO_2_ and photorespiration-derived CO_2_ originate from the same cellular compartment, i.e., mitochondria. Given this shared origin, it follows that CO_2_ released through mitochondrial respiration undergoes similar refixation dynamics as photorespiratory CO_2_ under low atmospheric CO_2_ concentrations (*C_a_*) and is comparably inhibited at elevated *C_a_*. The recovery efficiency and inhibition ratio of mitochondrial respiration-derived CO_2_ were consequently equivalent to those observed in photorespiration. To quantify *R_d_*, we therefore integrated the maximum mitochondrial respiration rate (*R_n_*) with the photorespiration-associated parameters *R_pe_-_i_* and *I_i_*, using the following calculation scheme:


(7) $$\begin{array}{c} R_{d-i}=R_{n-i}\times \left(1-R_{pe-i}\right)\;or\;R_{d-i}=R_{n-i}\times \left(1-I_{i}\right) \end{array}$$

where $R_{d-i}$ and $R_{n-i}$ represent mitochondrial respiration rates under light and dark conditions, respectively, at a given atmospheric CO_2_ concentration.

### 2.2 Study site and plants

The experiment was conducted at the Yucheng Comprehensive Experiment Station (36°50′N, 116°34′E; 20.3 m elevation) of the Chinese Academy of Sciences, located in the lower Yellow River basin. This semi-arid region exhibits a mean annual temperature of 13.4°C and receives 567 mm of precipitation annually, with 70% occurring between June and September (1985–2009 climate normals). The soil is classified as calcaric fluvisol (FAO-UNESCO system) with silt loam texture (12% sand, 66% silt, 22% clay; USDA classification) and pH 8.6 (Hou et al., 2012).

Wheat and bean were sown on 4 October 2013 and 3 May 2014, respectively. Field-grown plants experienced maximum photosynthetic photon flux density (PPFD) of 2000 μmol m^−^² s^−^¹ during sunny days. Measurements were conducted during key phenological stages: wheat from 12 ~ 25 May (characterized by 7 sunny days, predominantly cloudy skies, no effective precipitation, and a mean temperature of 29°C) and bean from 16 ~ 25 June (marked by predominantly cloudy conditions, 95 mm rainfall, and an average temperature of 30°C) 2014. We randomly sampled vigorous plants with homogeneous growth and measured the apical leaf of the fifth compound leaf (numbered from the base upward) on each seedling.

### 2.3 CO_2_ gas exchange measurement

Leaf-level CO_2_ exchange was quantified using a LI-6400XT portable photosynthesis system (LI-COR Biosciences, Lincoln, NE, USA) during two daily intervals (09:00 ~ 11:30 and 14:30 ~ 17:00). For each species, the leaves were acclimated in the cuvette for 15 min to stabilize gas exchange prior to measurements. Environmental conditions were maintained at leaf temperature 30 ± 0.3°C (wheat) or 33 ± 1.7°C (bean) with 60% relative humidity. Two sets of CO_2_ response curves were generated by systematically exposing leaves to a sequence of 12 atmospheric CO_2_ concentrations (C_a_: 0, 50, 80, 100, 150, 200, 380, 400, 600, 800, 1,000, and 1,200 μmol mol^−^¹). These experiments were conducted under two distinct oxygen conditions: ambient (21% O_2_) and low-oxygen (2% O_2_). For measurements of R_p_, R_d_, and other related parameters, a PAR of 2000 μmol m^−^² s^−^¹ was used. Conversely, a PAR of 0 μmol m^−^² s^−^¹ was employed to determine the mitochondrial respiration rates in the dark (Kang et al., 2014). The hypoxic gas mixture (2% O_2_) was supplied by Xinjian Air Plant (Yucheng, Shandong) and humidified via a 1.2 m³ buffer bag containing distilled water prior to entering the gas analyzer.

### 2.4 Statistics

Photosynthetic parameters were derived using Photosynthesis Assistant software (LI-COR Biosciences). Non-linear regression analyses implemented in SPSS 11.5 (IBM Corp., Armonk, NY, USA), based on Levenberg-Marquardt algorithm, including rectangular hyperbola model and Standard rectangular hyperbola model.

Treatment effects were assessed by two-way ANOVA with Tukey’s *post-hoc* test, while pairwise comparisons employed two-tailed Student’s t-tests (*p*< 0.05). All statistical visualizations were generated using GraphPad Prism 4.0c (GraphPad Software, San Diego, CA).

## 3 Results

### 3.1 Respiratory flux partitioning

At saturating irradiance (2000 μmol photons m^−^² s^−^¹), mitochondrial respiration (R_L-measured_) reached 6.548 ± 0.136 and 6.334 ± 0.342 μmol (CO_2_) m^−^² s^−^¹ in wheat and bean, respectively. R_d-measured_ exhibited the values of 2.036 ± 0.276 (wheat) and 1.893 ± 0.075 μmol (CO_2_) m^−^² s^−^¹ (bean). R_p-measured_ calculated via Equation 5 at zero CO_2_ (R_p-0-measured_) showed interspecific divergence, with wheat (4.511 ± 0.412 μmol (CO_2_) m^−^² s^−^¹) and bean (4.686 ± 0.274 μmol (CO_2_) m^−^² s^−^¹), we can find that there were significantly different between R_L-measured_ and R_p-measured_ (**Table 1**).

**Table 1:** Compared between measured and fitted values of R_L_, R_d_, and R_p-0_ for wheat and bean.

Measured		Wheat		Bean	
*R_L-measured_* (21% O_2_)		6.548 ± 0.136 a		6.334 ± 0.342 a	
*R_d-measured_* (2% O_2_)		2.036 ± 0.276 c		1.893 ± 0.075 c	
*R_p_*_-_ *_0-measured_*		4.511 ± 0.412 b		4.686 ± 0.274 b	
Fitted Models		*R_L-fitted_* (21% O_2_)		*R_d-fitted_* (2% O_2_)	
		Wheat	Bean	Wheat	Bean
*A*/*C_i_*	Biochemical model	21.067 ± 0.115^*^	17.600 ± 0.970^*^	6.333 ± 0.611^#^	4.700 ± 0.476^#^
	Rectangular hyperbola model	17.108 ± 0.978^*^	14.380 ± 0.680^*^	7.757 ± 1.155^#^	5.849 ± 1.283^#^
	Modified rectangular hyperbola models	14.172 ± 0.156^*^	12.713 ± 0.319^*^	5.599 ± 0.521^#^	4.612 ± 0.517^#^
*A/C_a_*	Biochemical model	20.667 ± 0.577^*^	6.850 ± 0.379^*^	20.200 ± 1.114^#^	2.467 ± 0.231^#^
	Rectangular hyperbola model	8.428 ± 1.100^*^	7.510 ± 0.545^*^	4.059 ± 1.277^#^	3.837 ± 0.493^#^
	Modified rectangular hyperbola models	7.353 ± 0.455^*^	7.102 ± 0.523^*^	2.716 ± 0.493^#^	2.779 ± 0.437^#^

The data represent the mean ± SD of five independent experiments; Measured values of *R_L_*, *R_p_* and *R_d_* for wheat and bean at 2000 μmol m^−2^ s^−1^ when the CO_2_ concentration was 0 μmol mol^−1^; Different letters at the same species indicate significant differences at the *P<* 0.05 level; ^*^ indicates that there are significant differences at the *P<* 0.05 level between the fitted values for *R_L-fitted_* and the measured values of *R_L-measured_* (21% O_2_). ^#^ indicates that there are significant differences of *R_d-measured_* at the *P<* 0.05 level between the fitted values and the measured values (2% O_2_).

### 3.2 Model performance evaluation

The three CO_2_ response models (biochemical, rectangular hyperbola, modified rectangular hyperbola) were evaluated by comparing R_L-fitted_ (21% O_2_) and R_d-fitted_ (2% O_2_) values with experimental measurements (**Table 1**).

Under the A/C_i_ framework, the modified rectangular hyperbola model showed the smallest deviations for both R_L-fitted_ and R_d-fitted_ compared to biochemical and rectangular hyperbola models. For example, R_L-fitted_ in wheat were 14.172 ± 0.156 μmol m^−^² s^−^¹ (modified model) versus 21.067 ± 0.115 (biochemical) and 17.108 ± 0.978 (rectangular), contrasting with measured values of 6.548 ± 0.136 (*P*< 0.05 for biochemical and rectangular models). Similar trends were observed for R_d-fitted_ (**Table 1**). Under the A/C_a_ framework, the modified model exhibited further accuracy improvements, particularly for R_d-fitted_. For wheat, A/C_a_-based R_d-fitted_ estimates (2.716 ± 0.493 μmol m^−^² s^−^¹) reduced deviations from measured values by 80.915% compared to A/C_i_ predictions (5.599 ± 0.521; Δ = 3.563 vs. 0.680 μmol m^−^² s^−^¹). In bean, A/C_a_ R_d-fitted_ errors decreased by 67.414% (Δ = 2.719 vs. 0.886 μmol m^−^² s^−^¹). R_L-fitted_ estimations under A/C_a_ also approached measured values more closely than under A/C_i_.

The modified rectangular hyperbola model under A/C_a_ framework demonstrated optimal consistency with experimental data, justifying its selection for subsequent analyses of respiratory responses to ambient CO_2_ (C_a_).

### 3.3 Photorespiration rate (R_p-measured_) responses to C_a_

**Figure 1:**
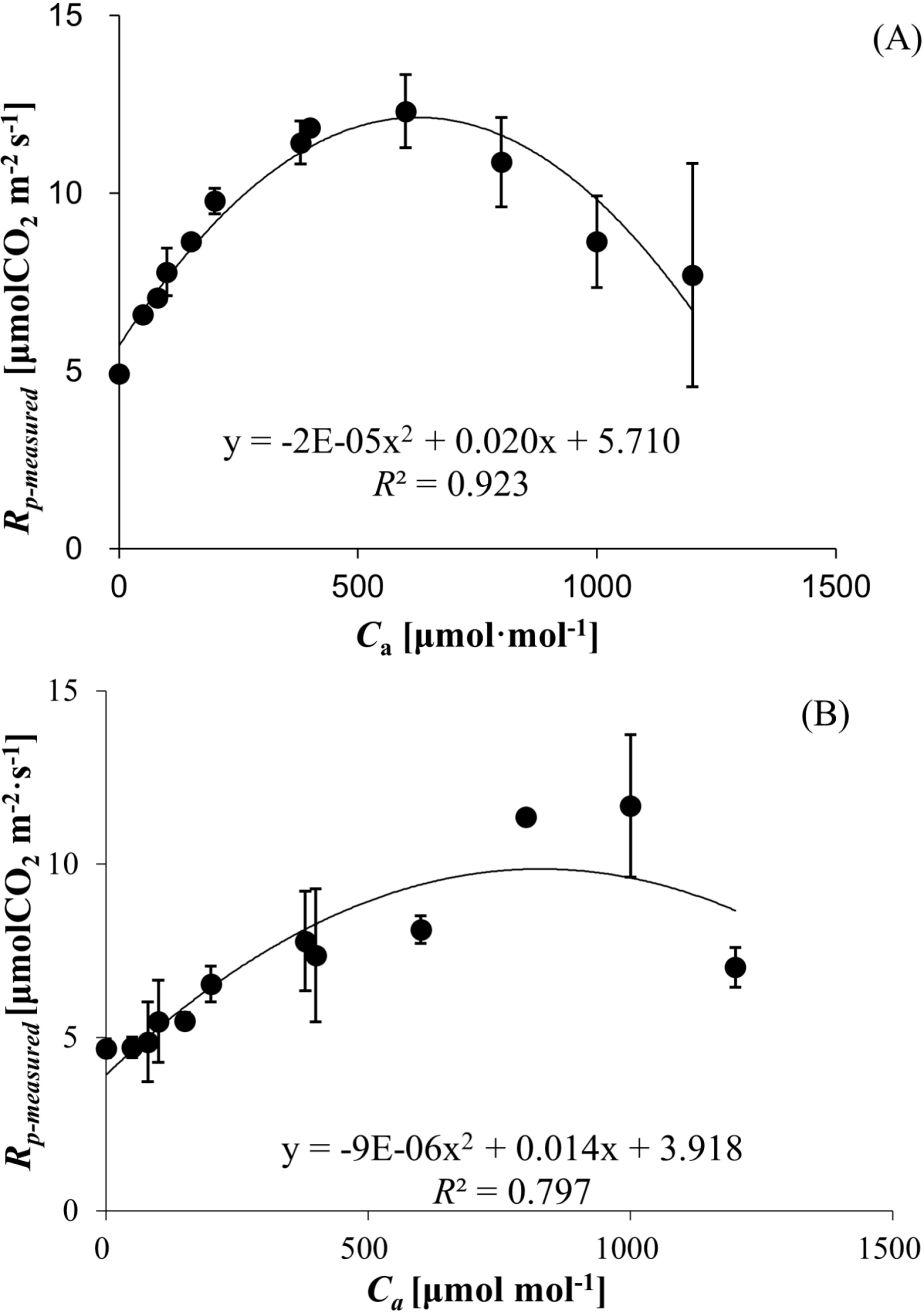
**(A)** Photorespiration rates (*Rp-measured*) of wheat responses to *C_a_*. **(B)** Photorespiration rates (*Rp-measured*) of bean responses to *C_a_*.

R_p-measured_ exhibited a unimodal relationship with ambient CO_2_ concentration in both wheat and bean, characterized by an initial increase followed by a decline at elevated C_a_ levels. The R_p-measured_ values ranged from 4.923 ± 0.171 to 12.307 ± 1.033 μmol (CO_2_) m^−^² s^−^¹ for wheat (**Figure 1A**) and 4.686 ± 0.274 to 11.673 ± 2.054 μmol (CO_2_) m^−^² s^−^¹ for bean (**Figure 1B**), with polynomial regression models demonstrating strong correlations (R² = 0.923 for wheat, R² = 0.797 for bean).

Crop-specific differences were evident in the C_a_ thresholds corresponding to peak R_p-measured_. Wheat achieved maximum R_p-measured_ (12.307 ± 1.033 μmol (CO_2_) m^−^² s^−^¹) at 600 μmol mol^−^¹ C_a_, whereas bean exhibited peak R_p-measured_ (11.673 ± 2.054 μmol (CO_2_) m^−^² s^−^¹) at 1000 μmol mol^−^¹ C_a_ (**Figures 1A, B**).

### 3.4 Mitochondrial respiration rate in the light (R_d-measured_) responses to C_a_

**Figure 2:**
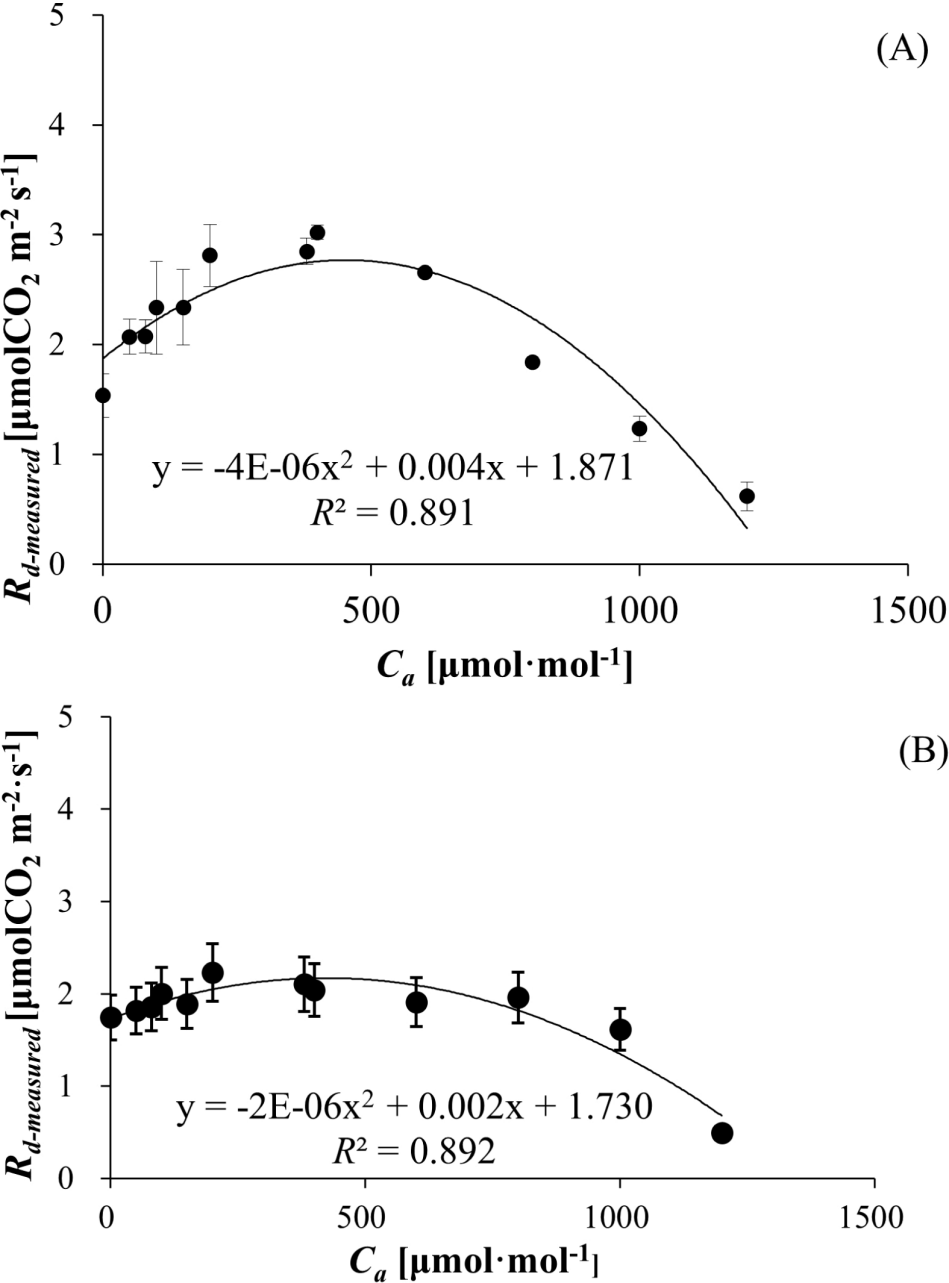
**(A)** Mitochondrial respiration rate in the light (*R_n-measured_*) of wheat responses to *C_a_*. **(B)** Mitochondrial respiration rate in the light (*R_d-measured_*) of bean responses to *C_a_*.

R_d-measured_, derived from Equation 7, exhibited a unimodal relationship with C_a_ for both species. The R_d-measured_ values ranged from 0.618 ± 0.131 to 3.021 ± 0.063 μmol (CO_2_) m^−^² s^−^¹ for wheat (**Figure 2A**) and 0.492 ± 0.069 to 2.323 ± 0.312 μmol (CO_2_) m^−^² s^−^¹ for bean (**Figure 2B**), with polynomial regression models demonstrating strong correlations (R² = 891 for wheat, R² = 0.892 for bean). R_d-measured_ initially increased to peak values of 3.021 ± 0.063 μmol (CO_2_) m^−^² s^−^¹ (wheat) and 2.323 ± 0.312 μmol (CO_2_) m^−^² s^−^¹ (bean), followed by declines at elevated C_a_. Polynomial regression confirmed robust correlations, reflecting C_a_-dependent modulation of R_d-measured_ dynamics.

### 3.5 Mitochondrial respiration in the dark (R_n-measured_ *)* responses to *C_a_* and O_2_ concentration

**Figure 3:**
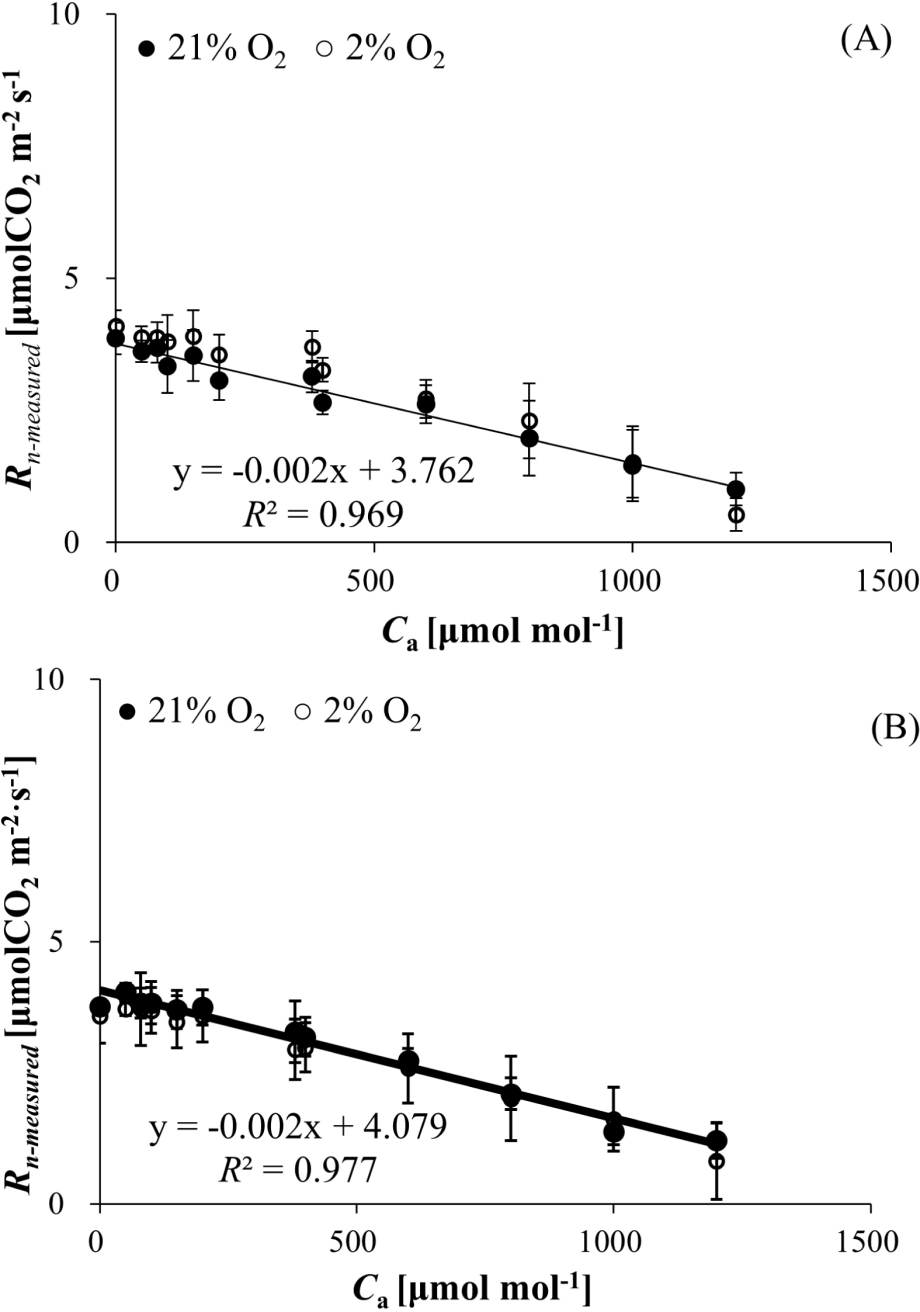
**(A)** Mitochondrial respiration rate in the dark (*R_n-measured_*) of wheat responses to *C_a_* and *O_2_* concentration. **(B)** Mitochondrial respiration rate in the dark (*R_n-measured_*) of bean responses to *C_a_* and *O_2_* concentration.

R_n-measured_ declined progressively with increasing C_a_ for both wheat and bean, independent of O_2_ levels (21% vs. 2%). At 21% O_2_, R_n_ spanned 1.453 ± 0.603 to 3.862 ± 0.557 μmol (CO_2_) m^−^² s^−^¹ (wheat, **Figure 3A**) and 1.210 ± 0.340 to 4.040 ± 0.167 μmol (CO_2_) m^−^² s^−^¹ (bean, **Figure 3B**). Under 2% O_2_, the ranges shifted to 1.512± 0.674 to 4.101 ± 0.297 (wheat) and 0.817 ± 0.607 to 3.718 ± 0.519 μmol (CO_2_) m^−^² s^−^¹ (bean). Strong negative correlations between R_n_ and C_a_ were observed (R² = 0.969 for wheat, R² = 0.977 for bean), with no significant O_2_ concentration effect (*P* > 0.05).

### 3.6 Recovery (R_pe−i_) and inhibition (I_i_) ratios response to C_a_

**Figure 4:**
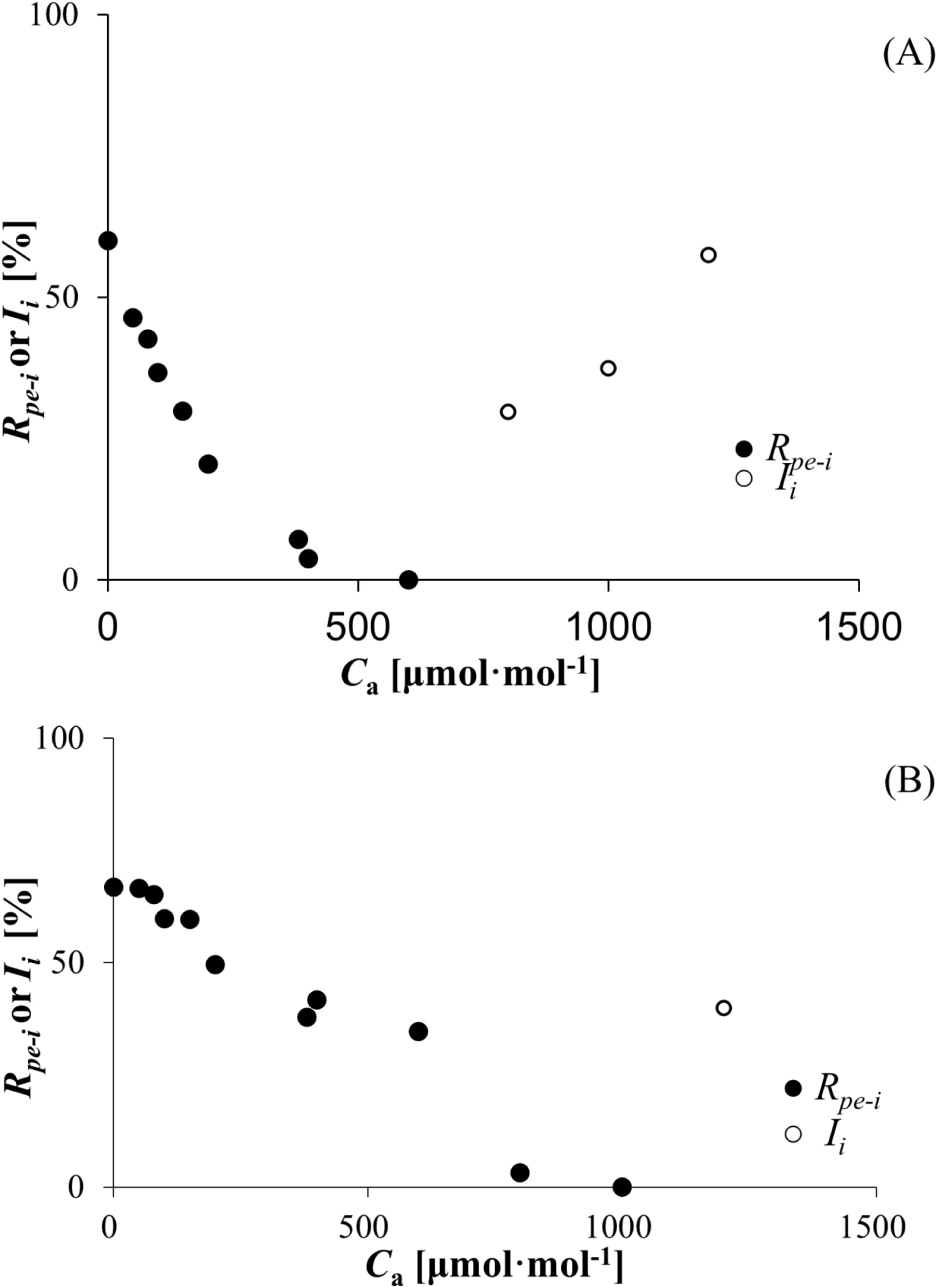
**(A)** Recovery and inhibition rates (*R_pe-i_* and *I_i_*) of wheat responses to *C_a_*. **(B)** Recovery and inhibition rates (*R_pe-i_* and *I_i_*) of bean responses to *C_a_*.

As mentioned before, 12.307 ± 1.033 and 11.673 ± 2.054 μmol (CO_2_) m^−2^ s^−1^ were the maximum photorespiration rate values for wheat and bean, respectively. On this basis, the CO_2_ recovery and inhibition ratios for photorespiration at different CO_2_ concentrations were estimated according to Equation 6. As C_a_ increased, the recovery ratios decreased from 59.995% (wheat) and 66.869% (bean) to zero, respectively (**Figure 4**). After that, the inhibition ratios increased sharply, reaching 57.456% (wheat) and 39.845% (bean), respectively.

## 4 Discussion

### 4.1 Respiratory flux partitioning

In the framework of traditional models, the overall respiration rate under light conditions (R_L_), which aggregates the rates of photorespiration (R_p_) and mitochondrial respiration in the light (R_d_), has frequently been either conflated with photorespiration alone (Zelitch, 1980; Ye and Yu, 2009; von Caemmerer, 2000) or has overlooked the significance of the reutilization of CO_2_ released during photorespiration (Kang et al., 2014). This conflation or oversight tends to result in a marked discrepancy between the observed photorespiration rates (R_p-fitted_) and their actual values. Our empirical findings, obtained under conditions of 21% O_2_ and atmospheric CO_2_ concentration of 0 μmol mol^−^¹, indicated that the R_L-measured_ values for wheat and bean (6.548 ± 0.136 and 6.334 ± 0.342 μmol m^−^² s^−^¹, respectively) significantly surpassed the accurately determined photorespiration rates (R_p-0-measured_ = 4.511 ± 0.412 and 4.686 ± 0.274 μmol m^−^² s^−^¹ for wheat and bean, respectively), as derived through the differential method (R_L-measured_ − R_d-measured_). This discrepancy underscores the systematic bias inherent in the traditional approach, which solely attributes R_L_ to photorespiration, thereby neglecting the distinct and crucial contribution of mitochondrial respiration under light conditions (R_d_).

Our analysis sheds light on the intricate dynamics between photorespiration and mitochondrial respiration within the context of photosynthesis, challenging the conventional understanding that has, until now, inadequately accounted for the nuanced contributions of these two processes. By distinguishing between R_L_ and its constituent components, R_p_ and R_d_, our study provides a more nuanced understanding of plant respiratory processes in the light, highlighting the significant role of R_d_. This clarification is pivotal for refining existing photosynthetic models, ensuring a more accurate representation of plant respiratory mechanisms and their implications for carbon metabolism.

### 4.2 Model performance evaluation

In the realm of plant physiology, accurately modeling the intricate processes of photorespiration and mitochondrial respiration under photosynthetic conditions is pivotal. Our study, by comparing measured values with the fitted values (**Table 1**), underscores the remarkable precision of the Modified rectangular hyperbola models, especially when employing A/C_a_ curves for estimating R_L-fitted_ and R_d-fitted_ values. This finding aligns with the observations made by Ye and Yu (2009), who posited that the discrepancies observed in earlier models could be attributed to the misrepresentation of intercellular CO_2_ concentrations by the C_i_ values used in those models.

However, a notable divergence persists between the fitted values generated by the A/C_a_ model and the actual measurements. This discrepancy led to the conclusion that previous models might have overlooked the significant impact of CO_2_ concentration on R_d_ and R_p_. Our analysis suggests an imperative need for further research aimed at refining these models to enhance their accuracy.

### 4.3 Photorespiration rate and Mitochondrial respiration rate in the light responses to C_a_

This study revealed a nonlinear regulatory mechanism of CO_2_ concentration on photorespiration rate (R_p-measured_) and mitochondrial respiration rate in the light (R_d-measured_) in C_3_ plants: R_p-measured_ increased with rising CO_2_ concentrations when external CO_2_ levels were below species-specific thresholds (600 μmol mol^−^¹ for wheat and 1000 μmol mol^−^¹ for bean), whereas exceeding these thresholds triggered a significant suppression of R_p-measured_. As for R_d-measured_, the thresholds were 400 μmol mol^−^¹ for wheat and 200 μmol mol^−^¹ for bean. This phenomenon can be explained by the dynamic interplay between RuBisCO enzyme activity and chloroplast microenvironmental conditions. At low CO_2_ concentrations, although the carboxylation activity of RuBisCO is globally constrained by substrate limitation (Caemmerer and Edmondson, 1986) and RuBP regeneration becomes impaired (Badger et al., 1984), the CO_2_ released during photorespiration is efficiently re-assimilated by photosynthesis due to its proximity to the chloroplast inner membrane (Yadav et al., 2020). This tight coupling between photorespiratory CO_2_ release and photosynthetic refixation (Häusler et al., 2002; Loreto et al., 1999; Busch et al., 2013; Kang et al., 2013) partially mitigates the inhibitory effects of low CO_2_ on the Calvin cycle. Concurrently, enhanced photosynthesis elevates chloroplast O_2_ levels (Sharkey, 1988), temporarily promoting RuBisCO oxygenation activity and driving the “paradoxical” increase in R_p_ and R_d_. However, when CO_2_ concentrations surpass species-specific thresholds, the chloroplast CO_2_/O_2_ ratio undergoes a fundamental reversal (Brooks and Farquhar, 1985), favoring RuBisCO carboxylation through competitive substrate inhibition of oxygenation, leading to a decrease in R_p-measured_ and R_d-measured_. The observed threshold divergence between wheat and bean likely arises from two interconnected mechanisms: First, interspecific variations in RuBisCO kinetics (e.g., CO_2_ affinity) and leaf anatomical adaptations regulating CO_2_ diffusion resistance, consistent with the multiscale regulatory complexity of photorespiratory metabolism (Wang et al., 2020; Celebi-Ergin, 2022). Second, differential cellular metabolic demands—for example, the approximately twofold higher CO_2_ threshold for peak photorespiration in bean (1,000 vs. 600 μmol·mol^−^¹ in wheat) aligns with the hypothesis proposed by Krmer et al. (2022) that elevated photorespiratory flux in legumes supports nitrogen assimilation-coupled amino acid synthesis. These findings not only provided theoretical support for crop-specific CO_2_ fertilization strategies in controlled-environment agriculture but also advance our understanding of carbon-oxygen metabolic homeostasis in C_3_ plants.

### 4.4 Mitochondrial respiration rate in dark (*R_n_*_-measured_) responses to C_a_ and O_2_ concentration

Accurately estimating the dark respiration rate of a plant facilitates the calculation of its maximum carboxylation rate, respiration rate in the light, electron flow partitioning, and other important photosynthetic parameters (Wang et al., 2001; Yin et al., 2011). Oxygen is essential for the respiration process in plant cells (Moseley et al., 2018). However, the results of our experiments showed that there was no significant difference in dark mitochondrial respiration rates between wheat and bean at 2% and 21% O_2_ (**Table 1**). It’s meant to be sufficient oxygen for mitochondrial respiration at 2% O_2_. In addition, we can notice that there is linear regulatory mechanism of CO_2_ concentration on R_n-measured_, i.e., the *R_n_* decreased as C_a_ increased (**Figures 3A, B**), which was different from the relationship between R_d-measured_, R_p-measured_, and carbon dioxide. We speculate that this may be due to the increase in CO_2_ concentration inhibiting the activity of certain enzymes related to dark respiration, and this effect is stronger than that of R_d-measured_ and R_p-measured_. Reuveni and Gale (1985) also obtained the similar results that CO_2_ concentration has a strong effect on dark respiration rates in plants.

## 5 Conclusion

This study advanced our understanding of respiratory parameter estimation in C_3_ plants by systematically evaluating the accuracy of photorespiration (R_p_) and mitochondrial respiration in the light (R_d_) derived from CO_2_-response models. Key findings revealed that the modified rectangular hyperbola model under the A/C_a_ framework outperformed traditional A/C_i_ models in estimating R_p_ and R_d_, yet significant discrepancies persisted between modeled and empirical values (*p*< 0.01), highlighting inherent limitations in current methodologies. Notably, CO_2_ concentration exhibited dose-dependent, non-linear regulation of respiratory parameters. R_p-measured_ in wheat and bean demonstrated unimodal responses to C_a_, peaking at 600 and 1,000 μmol·mol^−^¹, respectively, before declining due to competitive inhibition of RuBisCO oxygenation. Similarly, R_d-measured_ displayed different thresholds between bean and wheat (400 μmol mol^−^¹ for wheat and 200 μmol mol^−^¹ for bean.). The identification of strong polynomial correlations (R² > 0.89) between C_a_ and respiratory fluxes challenges conventional assumptions of linear responses, emphasizing the need to integrate CO_2_-responsive regulatory dynamics into photosynthetic models. Furthermore, dark respiration (R_n-measured_) exhibited a linear decline with rising C_a_, independent of O_2_ concentration, suggesting distinct mechanistic controls compared to light-dependent respiration.

These findings provide critical insights for refining photosynthetic models by incorporating CO_2_-mediated respiratory adjustments. The empirical relationships established here offer a framework for optimizing carbon assimilation strategies in crops under rising atmospheric CO_2_, particularly in controlled-environment agriculture.

## Data Availability

The datasets presented in this article are not readily available because data privacy. Requests to access the datasets should be directed to kanghuajing@126.com.
